# Miscellaneous Notices

**Published:** 1856-10

**Authors:** 


### MISCELLANEOUS NOTICES.

*American Dental Convention.*—It will be seen from the proceedings published in another part of the present number of the Journal, that the second meeting of the above named association was held at Hope Chapel, in the city of New York, on Wednesday, Thursday and Friday, the 6th, 7th and 8th of August, 1856, and we hesitate not to say, it was the most numerous assemblage of dentists ever convened either in this or any other country. Twenty states, the District of Columbia and the Island of Cuba were represented on the occasion, and there were present, as nearly as we could judge, at the morning session of the second day, about two hundred and twenty-five, though the Secretary only succeeded in obtaining the names of one hundred and eighty-six, but many of the members had left the city before he commenced taking them—this having been deferred until towards the close of the Convention.

The deliberations of this large assemblage were characterized by the utmost harmony and good feeling—the object of the members generally of the Convention appearing to have been a desire to enlarge their professional knowledge, that they might return to their respective fields of labor with increased ability for usefulness.

Among the subjects which came up for discussion before the Convention, as will be seen from the proceedings, and which occupied the greater portion of the time of its meetings, were, the pathological conditions of dentine, and the best preparation of gold for filling teeth. These discussions were highly interesting, and will, we have no doubt, be read with interest by the members of our profession. Professor Watt, of Cincinnati, Ohio, read, by request of the Convention, an interesting paper, which we hope to be able to lay before our readers in a future number, on the action of certain remedial agents on inflamed dentine. A paper was also read by Professor Townsend, of Philadelphia, on Professional Fees, which elicited the approbation of the members generally, and was ordered to be printed in pamphlet form.

During the afternoon session of the last day of the Convention, a number of improvements and inventions were presented, which, in a future number of the Journal, we may take occasion to notice at some length. We would do so now, if time permitted.

That the annual deliberations of this Convention will be productive of great good, does not, we think, admit of doubt, and most sincerely do we trust that the future meetings may be as largely attended as the last. The next is to be held in Boston, the second Tuesday in August, 1857.

On the evening of the second day, Messrs. Jones, White & McCurdy, gave a splendid entertainment at the Astor House, to the members of the Convention, and on the evening of the third day, they were again feasted with good things by the dentists of New York, Brooklyn and Williamsburg.

*Western Dental Society.*—We publish in another part of the present number of the Journal, the proceedings of a meeting of the Western Dental Society, held at Chicago, Ill., July 30, 1856, reported by Dr. Solyman Brown, for the Dental Forcep. The proceedings of this Association will also be found interesting as showing the efforts which the dentists of the West are making for the elevation and advancement of the profession. We had an invitation to attend and would most gladly have availed ourselves of it had circumstances permitted. We wish our brethren of the West great success in their praiseworthy enterprise, and most earnestly do we hope we may have the pleasure of being with them at some future meeting.

*Dental Obturator.*—This is the name of a spirited and ably conducted Quarterly, of some thirty-two pages, under the editorialship of John S. Clark, D. D. S., of New Orleans, and devoted to the science and art of dentistry. We had the pleasure of making the acquaintance of the editor at the Dental Convention in August last, and have since received from him the first volume of his valuable and interesting publication, neatly bound, for which, we beg to return him our sincerest thanks. We had previously, a short time before the publication of our July number, received a number of this neat little Quarterly, and intended to have called the attention of our readers to it, but it escaped our attention until it was too late to do so. We hope to receive it hereafter regularly, and take pleasure in placing it upon the list of our exchanges.

*British Journal of Dental Science.*—Some twelve or fifteen years ago we had the pleasure of congratulating our professional brethren of Great Britain on the advent, first of a quarterly, and soon after of a semi-monthly journal, devoted to the advancement of dental science. The first of these publications terminated, we believe, with the issue of one number; the second, a very ably conducted paper, was continued, we regret to say, but little more than a year, when it also ceased to exist. The members of the dental profession are now vastly more numerous than they were at the time these publications were started, and will, we doubt not, extend to the present enterprize, sufficient support, both in contributions to its pages and subscriptions, to ensure its permanent continuance. It is published monthly, and we have received the July and August numbers, which contain several interesting and well written articles, some of which we would copy in the present number of our journal, had we received them in time. The editorial of the second number sets forth very ably, the importance of the immediate organization of a college or institute of dental surgery in England, and we hope the wishes of the editor may soon be realized. We would be glad, if space permitted, to transfer his remarks upon this subject to the pages of our own journal, but at present we cannot do so.

The conductors of the British Journal of Dental Science have our best wishes for the success of their enterprize.

*Dental Register of the West.*—The July number of this valuable and ably conducted quarterly, contains the valedictory of our old friend, Professor Taylor, who was the original projector, and has been the principal, and a large portion of the time, the sole editor of the publication. Few men would persevere as he did, under the discouragements with which we know he had to contend, before sufficient support was given to the noble enterprize, in which he had engaged to indemnify him against actual loss. But these difficulties did not dampen the ardor of his efforts for a single moment, or cause him to falter in the undertaking; on the contrary, they only served to strengthen his energies, and thus he struggled on, increasing the value of the Register every year, until it was established upon a firm basis. It is impossible for any one not engaged in an enterprize of this kind to form an adequate idea of the difficulties and vexations with which the conductors of such publications, have, for a while, at least, to contend.

We regret most sincerely the loss, from the corps editorial, of so valuable a co-laborer. In retiring from the editorial duties of the Register, Professor Taylor takes with him our best wishes, and we almost envy him the enjoyment he must experience in being relieved from the toil and perplexity necessarily incident to the conducting of a professional periodical. We trust, however, he will not lay aside his pen altogether.

But while the retirement of Professor Taylor, from the editorship of the Register will be regretted by the readers of that publication generally, it cannot be othewise than gratifying, that it is confided to gentlemen in every respect so well qualified to conduct it, as Professors Watt and Taft, under whose editorial management, it will hereafter be carried on. In assuming the duties of the editorial chair, we most cordially extend to them the right hand of fellowship, and welcome them to their new field of labor.

*The New York Journal of Medicine.*—This excellent monthly comes to us in a new and improved form. It has incorporated with it the New York Medical Times, and has added to its former editorial corps, Dr. H. D. Bulkley, formerly editor of the Medical Times. In addition to the usual variety of papers on medical subjects, every number hereafter is to contain abstracts of the proceedings of the medical societies, as well as of those read before them, and clinical reports of the hospitals of the city Among other valuable papers, the present number contains one on the connection of bronzed skin, with disease of the super-renal capsules, illustrated with three colored engravings.

*Aluminium.*—This metal has been suggested to dentists as a substitute for gold in plate-work. It possesses many properties which would render it very desirable to employ it. In the first place its specific gravity (2.25) is much below that of the ordinary metals. It is nearly that of glass, and this lightness must of course add greatly to the comfort with which plates made of the new metal could be worn. Again, it has no tendency to rust and is not acted on by any of the ordinary solvents. Sulphur does not affect it, nor will any of the oxy-acids dissolve it, its only solvent being hydrochloric acid. This indifference to chemical agents has been supposed to be due to the formation of an oxyd, a thin film of transparent sapphire, at the instant it comes in contact with the atmosphere. Its fusing point is lower than that of silver, and higher than the melting point of zinc. It is malleable, ductile and sonorous.

Its present price in England, is £3, or fifteen dollars per ounce. This is about 25 per cent. cheaper than gold. In reality, however, the ratio between its price and that of the precious metal now used for plate-work, is much less than this statement would imply. Being so very far below gold, in specific gravity, a given weight of aluminium would go more than eight times as far as the same quantity of gold.

With most of the metals it forms characteristic alloys. With copper it forms a fine gold-colored alloy which does not appear to tarnish readily. Mixed with gold, a very small proportion of it changes the yellow color of that metal to gray.

For the benefit of those who may wish to experiment with this new metal, we give the formula for a *solder of aluminium.* It is composed of *two* parts of aluminium and *one* of silver—without flux.

*Saliva.*—M. Longet has been examining the relations of *sulpho-cyanide of potassium.* He confirms the opinion of Wright, Lehmann and other chemists that this salt is a constant ingredient of saliva, and attributes the variations in its quantity solely to the degree of concentration of the salivary fluid. It exists not only in the mixed saliva but in that of each individual gland.

